# Can we use the pharmacy data to estimate the prevalence of chronic conditions? a comparison of multiple data sources

**DOI:** 10.1186/1471-2458-11-688

**Published:** 2011-09-05

**Authors:** Francesco Chini, Patrizio Pezzotti, Letizia Orzella, Piero Borgia, Gabriella Guasticchi

**Affiliations:** 1 Agency of Public Health, Lazio Region; via di S. Costanza 53, 00198 Rome, Italy

## Abstract

**Background:**

The estimate of the prevalence of the most common chronic conditions (CCs) is calculated using direct methods such as prevalence surveys but also indirect methods using health administrative databases.

The aim of this study is to provide estimates prevalence of CCs in Lazio region of Italy (including Rome), using the drug prescription's database and to compare these estimates with those obtained using other health administrative databases.

**Methods:**

Prevalence of CCs was estimated using pharmacy data (PD) using the Anathomical Therapeutic Chemical Classification System (ATC).

Prevalences estimate were compared with those estimated by hospital information system (HIS) using list of ICD9-CM diagnosis coding, registry of exempt patients from health care cost for pathology (REP) and national health survey performed by the Italian bureau of census (ISTAT).

**Results:**

From the PD we identified 20 CCs. About one fourth of the population received a drug for treating a cardiovascular disease, 9% for treating a rheumatologic conditions.

The estimated prevalences using the PD were usually higher that those obtained with one of the other sources. Regarding the comparison with the ISTAT survey there was a good agreement for cardiovascular disease, diabetes and thyroid disorder whereas for rheumatologic conditions, chronic respiratory illnesses, migraine and Alzheimer's disease, the prevalence estimates were lower than those estimated by ISTAT survey. Estimates of prevalences derived by the HIS and by the REP were usually lower than those of the PD (but malignancies, chronic renal diseases).

**Conclusion:**

Our study showed that PD can be used to provide reliable prevalence estimates of several CCs in the general population.

## Background

One of the most important aim of public health is to provide an accurate evaluation of the population health conditions, its need for care and related costs.

Usually, the estimate of prevalence for the most common chronic conditions (CCs) is calculated using direct methods such as prevalence surveys [1] but also indirect methods using health administrative databases that collect this information for other reasons were used [2].

Ideally, prevalence surveys that estimate the prevalence of CCs by a clinical evaluation, and not only by self-reported information from subjects should be performed. However, they are expensive and when performed were limited to elderly and in specific geographical areas [3,4].

Prevalence surveys based on self-reported information are regularly conducted in several countries to provide estimates for several CCs [1,5]. Some of these surveys present the advantage to be not particularly expensive but, at the same time, they are criticized because the presence/absence of the disease is self-referred and thus conditioned by potential bias. Furthermore, these surveys refer to a sample of the population and thus are also limited by the sampling uncertainty. In particular, these estimates could be biased because some individuals likely might not be reached by the survey (e.g., very old people living in retirement homes).

As far as the use of health administrative databases to estimate the prevalence of some diseases, hospital discharge registries are those more often used because they collect specific information about diagnoses [6]. However, in some cases the accuracy of diagnostic code can be low [7,8]; furthermore, for some diseases the probability of being hospitalized, also for a long period, is very low and thus it might underestimate the actual prevalence.

The health administrative database of the general practitioners (GPs) has also been used to estimate prevalence given that for some conditions it is likely that a subject with the studied disease may be in charge of the GPs [9,10]. However, GPs are not formally requested to collect specific databases with information about diseases and they collect data quite exclusively for facilitating their routine management such as drug prescriptions, doctor's notes, et cetera. This means that the quality about diagnosis may be heterogeneous; furthermore, for some CCs, GP has likely very few contacts with the patients; finally, at least for Italy, the access by public health services to GP's databases is impossible given that there are no statutory compliances for that.

Recently, the use of drug prescription database has been proposed to estimate the prevalence of specific CCs [11,12]. This can be done when the drug prescriptions are unambiguously used for the treatment of these diseases (e.g., insulin for diabetes mellitus). In Italy drug prescriptions are collected at regional level and the coverage is expected to be extremely high because they are used for reimbursement by the regional health service (RHS).

The objective of this study is to provide estimates of prevalences of people diagnosed with several CCs in Lazio region, Italy, in 2006 using the drug prescription's database and to compare these estimates with those obtained using other health administrative databases. These prevalence estimates were also compared, when possible, with that reported by the survey performed in 2004-2005 by the Italian bureau of census (ISTAT) [1].

## Methods

### Context

All Italian citizens are enrolled in the National Health Service (NHS) [13-15] which provides health care free of charge. This entails that for administrative reasons several registries collecting information on use of health services reimbursed by each regional HS are needed. As far as our objective, there are three administrative archives of interest: one contains all outpatient drug prescriptions; another contains all the citizens exempts for the health expenses because affected by important diseases; the third one collects all discharges from hospitals.

### Setting

Lazio is a region of central Italy (including Rome) with a population of around 5,300,000 at the end of 2006 census [16] and, as well as all the other Italian regions, it provides its citizens with a universal coverage for health care.

### Data sources

#### Regional informative system on drugs (pharmacy data)

The Italian National Health System (NHS) provides medications to the population through the National Therapeutic Formulary (NTF) [17]. Lazio region has an informative system collecting all relevant data (i.e., patients' demographics information, the tax code, drug code, dose, formulation, number of packages, date of prescription) about prescribed drugs by GPs and public ambulatories, belonging to a list called "drugs in class A" of the NTF. Drugs for CCs treatments might be totally or partially reimbursed by the RHS and are often subject to restrictive note (in Italian called "Nota CUF") for dispensing defined by the Italian Medicine Agency (AIFA - Agenzia Italiana Farmaco) [18]. These restrictions can be considered as guidelines for a more appropriate use of pharmaceuticals. The "Nota CUF" defines the CC for dispensing the drug and increased our ability to capture drug users affected by the selected CCs.

Drugs are classified by ATC groups, according to the World Health Organization (WHO) Anatomical Therapeutic Chemical (ATC) classification system [19].

Drugs dispensed directly by the hospitals are not included in this informative system.

#### Regional Hospital informative system (HIS)

All hospitals are required to record data on standardized form about admission and discharge dates, patients demographic data (i.e., date of birth, gender, name, surname, municipality of residence, nationality, tax code), the principal diagnosis and up to five secondary diagnoses [coded by the International Classification of Diseases - ninth revision (ICD-9)], diagnostic procedures (also coded by the ICD-9), and death, if occurred during the hospitalization.

#### Registry of exempt patients from health care cost for pathology (REP)

The RHS requires that for some CCs it is needed to have a recognized diagnosis by the local health unit for having free access to health care services (e.g., drugs, laboratory and diagnostic visits). There is a regional registry containing demographic data of the patients with these diseases, the reason for requiring exemption, and the date of request for exemption.

#### ISTAT Health survey 2004-2005

This survey considered a probabilistic sample of more than 50,000 Italian families (3096 families and 7322 subjects in Lazio region). Using a face-to-face standardized questionnaire, it evaluated several aspects of health including the awareness of being affected by one or more CCs for the non-institutionalized population. The questionnaire is divided in several sections: health conditions, drug consumption, prevention, life styles and use and opinion of health services. The survey provided estimates prevalence rates of selected diseases [1].

#### Identifying individuals with CCs through pharmacy data

To detect subjects with specific CCs, we selected only those with at least one prescription of a drug unambiguously used for the treatment of that CCs. More specifically, for each CCs we referred to those ATC codes already proposed for the Italian context in another study (table 1) [11,12,20]. To limit potential unforeseen short-term use, we restricted our selection to individuals who had prescribed at least one drug belonging to the specific list of drugs identifying the CCs and with at least three packages during the year 2006.

**Table 1:** Chronic conditions (CCs), associated medications and ATC codes.

Chronic Disease	Drug Descriptions	ATC Codes
Alzheimer's	Anticholinesterase agents	N06DA; N06DX

	Digitalis glycosides, antiarrhythmics, diuretics, beta-adrenergic	C01AA; C01BA; C01BB; C01BC; C01BD; C01DA;
Cardiovascular diseases	blockers, alpha blockers, ACE inhibitors, calcium channel	C02AB; C02AC;C02CA; C03AA; C03BA; C03CA;
	blockers, antihypertensive vasodilators	C03CX; C03DA; C03EA; C03EB; C07AA; C07AB;

Chronic hepatitis	Interferons*	L03AB

Chronic renal disease	Agents for hyperkalaemia and hyperphosphataemia	V03AE; B03XA

Chronic respiratory illnesses	Inhaled corticosteroids, beta-2-adrenoreceptor agonist,	R03AC; R03AK; R03BA; R03BB; R03BC; R03CC;
	xanthines, leucotriene antagonists, cromolyn	R03DA; R03DC

Cirrhosis	Blood substitutives and plasmatic protein fractions*	B05AA; B02BA

Crohn's and ulcerative colitis	Intestinal corticosteroid agents	A07EA; A07EC

		A10AB; A10AC; A10AD; A10AE; A10BA; A10BB;
Diabetes	Insulins, biguanides, sulphonylureas	A10BD; A10BG; A10BX

		N03AA; N03AB; N03AD; N03AE; N03AF; N03AG;
Epilepsy	Anticonvulsivant barbiturates and congeners	N03AX

	Sympaticomimetic agents*, parasympaticomimetic	
Glaucoma	agents, anhydrase inhibitors*, ophthalmic beta blockers	S01EA; S01EB; S01EC; S01ED; S01EE; S01EX

HIV/AIDS	Nucleosides and nucleotides*, reverse transcriptase inhibitors	J05AB; J05AD; J05AF

		A04AA; H01CB; L01AA; L01AB; L01AX; L01BA;
		L01BB; L01BC; L01DB; L01XB; L01XX; L02AB;
Malignancies	Antineoplastics, 5HT3 antagonists*	L02AE; L02BA; L02BB; L02BG; L03AA; L03AX;
		R05DA; R05DB27

Migraine	Ergot alkaloids, 5HT1 agonists	N02CA; N02CC; N02CX

Paget's disease/other		H05AA02; H05AA03; H05BA; M05BA; M05BB;
osteoporosis chronic	Bisphosphonates*, calcitonin*	G03XC01; M05BX; A12AA; A12AX
conditions		

Parkinson's	Dopamine, MAO b inhibitors	N04AA; N04AB; N04BA; N04BC; N04BX

Psoriasis	Oral and topical antipsoriasis agents	D05AX; D05BB

		N05AA; N05AB; N05AC; N05AD; N05AF; N05AG;
Psychiatric disorders	Antidepressants, antipsychotics agents	N05AH; N05AL; N05AN; N05AX; N05BA; N05AA;
		N06AB; N06AX

		M01AB; M01AC; M01AE; M01AG; M01AH; M01AX;
Rheumatological conditions	Anti-inflammatory non-steroids*, gold salts, aminoquinolines	M01CB; M01CC; P01BA

Thyroid disorders	Thyroid replacement, antithyroid agents	H03AA; H03BB; H03BC

Tuberculosis	Antituberculosis antibiotics*, isoniazid	J04AB; J04AC; J04AK; J04AM

*drug subject to 'Nota' CUF (see methods section)

#### Identifying individuals with CCs in the HIS

We identified subjects with CCs using ICD9-CM diagnosis coding and we used the list proposed by Romano et al. [21]. Given that the probability to be recovered with some of the CCs in one year could be low, we referred to hospital discharges in Lazio region reported in the period 2002-2006 and not only to the year 2006 as done for the other sources.

#### Prevalence estimates

For each source used, subjects with each specific CCs were counted using a anonymous code (a string that is a unique identifier for each individual). To estimate prevalence of CCs obtained with the different data sources we used as denominator the population living in Lazio region as estimated at 1/1/2007 by the bureau of census [16]. We also provided estimates stratified by sex. Analyses were performed using SAS 9.2 and STATA 11.0. Regarding estimates from the ISTAT survey (clustered-two stage-sample of Italian families stratified by municipality), prevalence of chronic conditions and 95% confidence intervals (95%CI) were calculated using the "svyset" (defines the survey design for dataset) and "svytab" (calculates the absolute and the relative frequency taking into account the survey data) STATA commands [22].

## Results

From pharmacy data we identified drugs specific for 20 CCs (table 1). In 2006, about 2.5 million people (48% of the entire population) had prescribed reimbursed drugs for one or more of these CCs; these people were about the 72% of the individuals who had prescribed at least one drug reimbursed by the regional health system.

All drugs identifying the 20 CCs represented 61% of the entire volume of packages. Drug expenditure for these 20 CCs was about eight hundred million of euro, corresponding to 57% of the total expenditure (data not shown).

**Figure 1:**
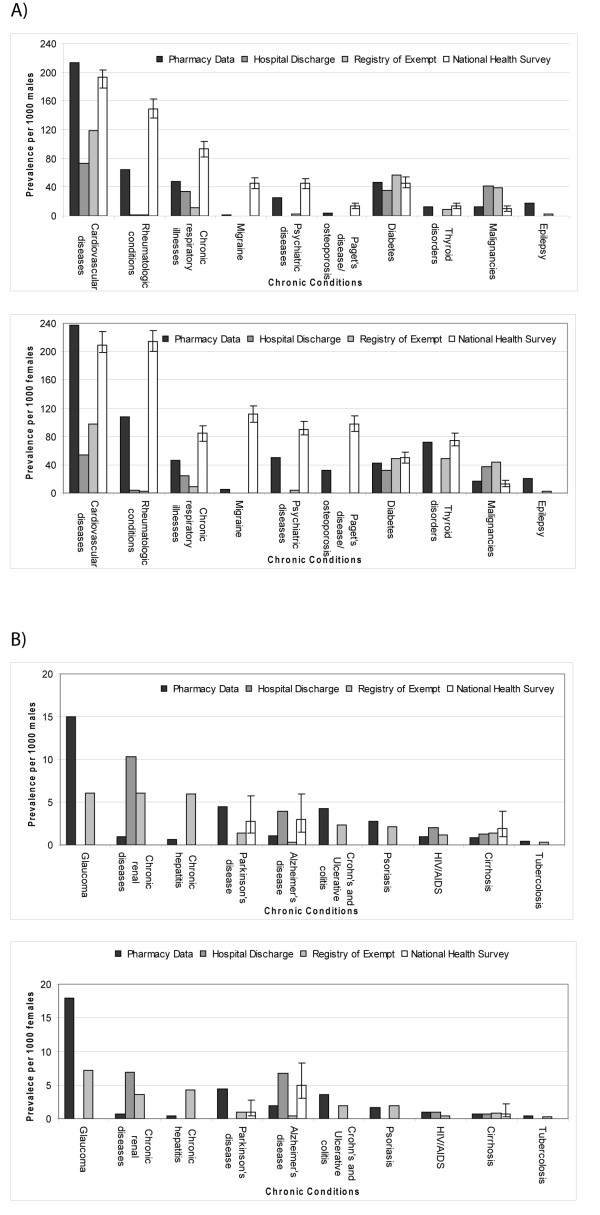
**(A-B): Estimates of prevalence per 1000 by sex for 20 chronic conditions (CCs) using different sources**. Lazio region, Italy.

Table 2 shows the number of subjects, the estimated prevalences, the volumes of prescriptions, the mean annual cost per individual for treatment, and the total cost. About 23% of the population received a drug for treating a cardiovascular disease, 9% for treating a rheumatologic conditions and then diminishing for other CCs. Table 3 compares the estimates of prevalences of the 20 CCs identifiable by the pharmacy data with those estimated by the ISTAT survey of 2005 and those obtained using the HIS and the REP, while the Figure 1(A) and 1(B) show the estimates of prevalences by sex using the four different sources.

**Table 2:** Number of individuals with identified 20 chronic conditions (CCs), reimbursed prescribed drugs and associated costs.

				Cost per	
		Prevalence		patient	Total Cost
Chronic Condition	Individuals	(per 1000)	Packages	(€)	(€)
Cardiovascular diseases	1199325	226.1	35,052,888	323.8	388,332,400
Rheumatologic conditions	463439	87.4	3,544,925	62.6	28,991,584
Chronic respiratory illnesses	251500	47.4	3,093,147	315.0	79,233,729
Diabetes	234117	44.1	4,565,308	192.6	45,100,938
Thyroid disorders	232852	43.9	1,906,099	23.5	5,477,555
Psychiatric diseases	202575	38.2	2,832,875	289.5	58,636,881
Epilepsy	99997	18.9	1,836,803	262.6	26,261,613
Paget's disease/osteoporosis chronic conditions	97167	18.3	991,775	262.8	25,532,022
Glaucoma	87793	16.5	1,571,032	229.2	20,120,303
Malignancies	77791	14.7	1,008,838	1,073.0	83,468,370
Parkinson's disease	23836	4.5	634,129	579.2	13,805,687
Crohn's and Ulcerative colitis	20763	3.9	384,769	416.5	8,647,390
Migraine	20354	3.8	349,518	382.9	7,794,343
Psoriasis	11735	2.2	156,922	180.1	2,113,543
Alzheimer's disease	8220	1.5	93,896	1,174.8	9,656,930
HIV/AIDS	5170	1.0	54,470	703.1	3,635,284
Chronic renal diseases	4271	0.8	60,225	2,218.3	9,474,249
Cirrhosis	3989	0.8	87,385	443.1	1,767,496
Chronic hepatitis and selective malignancies	2633	0.5	77,672	6,058.6	15,952,232
Tuberculosis	2093	0.4	32,552	113.3	237,101

Lazio region, Italy.

**Table 3:** Estimates of prevalence per 1000 for 20 chronic conditions (CCs) using different sources.

Chronic Condition	Prevalence Pharmacy Data 2006 (a)	Prevalence Hospital Discharge 2002-06 (b)	Prevalence Registry of Exempt 2006 (c)	National Health Survey 2005 (d)	95% CI	Relative difference between pharmacy data and other sources (%) #
Cardiovascular diseases	226.1	62.9	107.6	201.4	190.8 - 209.1	-10.9
Rheumatologic conditions (inflammations, arthritis, etc.)	87.4	2.6	1.7§	183.3	173.8 - 193.3	109.8
Chronic respiratory illnesses (asthma, COPD, etc.)	47.4	28.8	10.1	88.9	82.5 - 95.5	87.5
Migraine	3.8	n.a.	n.a.	79.7	72.9 - 87.0	1977.2
Psychiatric diseases	38.2	n.a.	3.0*	69.0	63.1 - 74.8	80.7
Paget's disease/other osteoporosis chronic conditions	18.3	n.a.	0.1**	57.3	51.8 - 63.4	212.8
Diabetes	44.1	34.0	52.6	48.0	42.9 - 53.7	19.2
Thyroid disorders	43.9	n.a.	29.4	45.4	40.4 - 51.0	3.4
Malignancies	14.7	39.1	42.0	11.4	8.9 - 14.5	186.1
Epilepsy	18.9	n.a.	2.5	n.a.		-87.0
Glaucoma	16.5	n.a.	6.7	n.a.		-59.7
Chronic renal diseases	0.8	8.6	4.8	n.a.		968.2
Chronic hepatitis and selective malignancies	0.5	n.a.	5.1	n.a.		922.3
Parkinson's disease	4.5	n.a.	1.2	1.9	1.0 - 3.4	-57.7
Alzheimer's disease	1.5	5.4°	0.4	4.0	2.7 - 6.0	158.1
Crohn's and Ulcerative colitis	3.9	n.a.	2.1	n.a.		-45.6
Psoriasis	2.2	n.a.	2.0	n.a.		-7.7
HIV/AIDS	1.0	1.5	0.8	n.a.		53.9
Cirrhosis	0.8	1.0	1.1	1.3	0.7 - 2.4	76.9
Tuberculosis	0.4	n.a.	0.3	n.a.		-19.5

Lazio region, Italy.

NA: not available or not estimable; °any type of dementia; § only rheumatoid arthritis; *excluded anxiety and depression; **only Paget's disease; ^# ^[(max(b,c,d)-a)/a

The estimated prevalences using the pharmacy data were usually higher than that obtained with one of the other sources. For thirteen CCs at least one of the other sources provided higher estimates; using an arbitrary cut-off of +15% in the relative difference between pharmacy data estimates and the maximum of the estimates from the other sources (last column of table 3), for 12 CCs the estimates were more greater than those obtained using the pharmacy data. It is of note that for 11 CCs the relative difference was higher more than 50% of that obtained with the pharmacy data.

Regarding the comparison with the ISTAT survey it is of a good agreement for cardiovascular disease, diabetes and thyroid disorder whereas for seven CCs (i.e., rheumatologic conditions, chronic respiratory illnesses, psychiatric diseases, Paget's disease or other osteoporoses, migraine, Alzheimer's disease, and cirrhosis) the prevalence estimates were 70% or more higher than those obtained by the pharmacy data. Estimates of prevalences were usually lower than those of the pharmacy data when derived by the HIS (but malignancies, Alzheimer's disease, chronic renal diseases, HIV/AIDS and cirrhosis) and by the REP (but malignancies, chronic renal diseases, and chronic hepatitis and selective malignancies). Also stratifying by sex there were similar results.

## Discussion

This study evaluated the possible use of pharmacy data on identifying individuals with several CCs in Lazio region, Italy.

We found that the highest prevalences of people diagnosed with CCs were for cardiovascular diseases and rheumatologic conditions. These results are coherent with previous estimates in analogous studies performed in another Italian region and in US [10,23]. The approach of measuring the prevalence of CCs using pharmacy data provided reliable estimates for diseases particularly impacting the health and social services such as Parkinson and Alzheimer disease. Prevalence estimates of these diseases using pharmacy data were comparable to those found in other European studies [24-26].

Data were then compared in terms of prevalence with other health administrative databases and with prevalence estimates obtained by the ISTAT survey. Assuming that all sources correctly identified each specific CCs, we observed that for several of these CCs the pharmacy data was better on identifying cases. This was particularly pronounced with respect to the HIS and to the REP that only in few cases provided higher estimates than the pharmacy data. With respect to the prevalence estimates by the ISTAT survey we highlighted that for some CCs the prevalences estimated by the pharmacy data had a quite good level of agreement (i.e., cardiovascular diseases, diabetes, thyroid disorders, malignancies, cirrhosis). For rheumatologic conditions, chronic respiratory illnesses, psychiatric diseases, osteoporosis, migraine the agreement was very low and the ISTAT survey provided extremely higher estimates of prevalences compared to those obtained with the pharmacy data.

One possible explanation for this discrepancy between pharmacy data and ISTAT survey is that the latter measured the self reported CCs and several studies suggested that the accuracy of self-reporting can be low for some CCs. As an example, it has been shown that subjects over-report rheumatologic conditions in surveys where the diagnosis is self-reported [27,28]. Furthermore, self-reporting accuracy is likely to be very low for CCs with a vague definition such as migraine. Otherwise, it is likely that those treated (and then identified with pharmacy data) are likely to refer to a more severe case definition.

The HIS is likely less sensitive because it identifies only the more severe cases that need hospitalization. Also the REP is likely less sensitive due to the fact that the free access to health care services is also given to citizens belonging to specific groups of age (e.g., people aged ≥ 65 years old) or of low income and thus there is no practical reason in some cases to require the exempt for a specific CCs.

Some CCs prevalences were comparable with those estimated by an Italian health administrative database of GPs [10]. For this source there were no available data of CCs at regional level, but our findings showed a good agreement with some Italian prevalence of CCs, particularly with diabetes [29] and chronic obstructive pulmonary disease [10].

The prevalence estimates obtained by pharmacy data have several advantages compared to those obtained by other health administrative databases and by cross-sectional surveys.

The prevalence estimates can be easily obtained and they provide estimates not conditioned by sampling problems.

In particular, these estimates can be provided also by very small geographical areas while this is not possible in surveys planned to provide reliable information, in terms of precision, only at a national or regional level. Furthermore, these estimates can be updated frequently. Another advantage is that the ATC coding used to identify CCs is internationally used and this allows immediate comparisons of prevalence estimates in other countries. Finally, it is important to highlight that pharmacy data could also be used to evaluate the incidence for some acute diseases in case a specific treatment would be available.

## Limitations

The present study has several limitations. All the health databases used are affected by selection bias because they likely do identify more severe cases. Statistical techniques had been proposed to correct for the selection bias using external information [30] such as health surveys but in our case this approach was not feasible because the non-availability of survey data. Another approach to correct for selection bias is using capture-recapture techniques [31] but we were not authorized to link the health databases due to privacy reasons.

Using pharmacy data to identify a specific CC implies that those drugs are used exclusively for the treatment of that CC. Furthermore, it is also important that the drug identifying CCs be used in any stage of the disease. We feel that for some CCs the coverage of drug treatment is low and hence a poor proxy for prevalence (i.e., anticholinesterase agents for dementia, interferons for chronic hepatis B, drugs listed for chronic renal disease, for cirrhosis, and for malignancies). It is also important to remind that for some diseases, such as osteoporosis and diabetes, the pharmacological treatment is not given mainly because of under-diagnosis of the conditions.

Another limit regards the potential inclusion of individuals without the specific CC evaluated who used the drug as incidental users or for other CCs not considered in this study. However, this limit has had little impact because we referred to drugs that had restrictive notes for dispensing, restricting the use only to individuals diagnosed with that CCs (see methods section). Furthermore, we included only individuals who had prescribed three or more packages of drug used to identify the CCs, but no sensitivity analysis was performed to determine if increasing the number of packages resulted in substantial changes in the prevalence estimates.

Finally, this study did not consider drugs directly prescribed/administered in hospital setting.

## Conclusions

Our study showed that pharmacy data can provide, in several cases, reliable prevalence estimates of CCs in the general population. The estimates obtained could be a quick and priceless alternative to survey data that assess the health population status.

The methodology offers the possibility of international comparison of disease prevalence, prescribing and drug costs in managing CCs.

## Competing interests

The authors declare that they have no competing interests.

## Authors' contributions

FC developed the concept, collected the data, participated in the analysis, and initiated the initial and subsequent drafts. PP participated in the analysis, and substantially revised the manuscript drafts. LO provided substantial methodological comments on the drafts.

PB and GG contributed to the conception of the research question, assisted in revising the manuscript. All authors reviewed and approved the final manuscript.

## Pre-publication history

The pre-publication history for this paper can be accessed here:

http://www.biomedcentral.com/1471-2458/11/688/prepub
